# Study Partners Recommendation for xMOOCs Learners

**DOI:** 10.1155/2015/832093

**Published:** 2015-01-12

**Authors:** Bin Xu, Dan Yang

**Affiliations:** ^1^Computing Center, Northeastern University, Shenyang 110819, China; ^2^College of Information Science and Engineering, Northeastern University, Shenyang 110819, China

## Abstract

Massive open online courses (MOOCs) provide an opportunity for people to access free courses offered by top universities in the world and therefore attracted great attention and engagement from college teachers and students. However, with contrast to large scale enrollment, the completion rate of these courses is really low. One of the reasons for students to quit learning process is problems which they face that could not be solved by discussing them with classmates. In order to keep them staying in the course, thereby further improving the completion rate, we address the task of study partner recommendation for students based on both content information and social network information. By analyzing the content of messages posted by learners in course discussion forum, we investigated the learners' behavior features to classify the learners into three groups. Then we proposed a topic model to measure learners' course knowledge awareness. Finally, a social network was constructed based on their activities in the course forum, and the relationship in the network was then employed to recommend study partners for target learner combined with their behavior features and course knowledge awareness. The experiment results show that our method achieves better performance than recommending method only based on content information.

## 1. Introduction

Massive open online courses (MOOCs) provide free learning opportunities for worldwide people. The term “MOOC” is defined as a course that is open with no barriers to entry, neither cost nor education criteria. The courses are also online, accessed on the Web, and are massive, requiring a significant number of students to contribute to a connected learning environment. Currently, there are two different types of MOOCs: cMOOCs and xMOOCs. In 2008, George Siemens and Stephen Downes opened up a course called Connectivism and Connective Knowledge Online Course (CCK08). It is the first cMOOC course. The “c” in cMOOC stands for connectivist. Based on the idea that learning happens within a network, where learners use digital platforms such as blogs, wikis, social media platforms to make connections with content, learners can create and construct knowledge by themselves. However, the xMOOCs such as* Coursera* (http://www.coursera.org/),* edX* (http://www.edx.org/), and* Udacity* (http://www.udacity.com/) platforms have grabbed the spotlight from 2012 even though critics of xMOOCs argue that xMOOCs are inferior to the university courses they mimic because they eliminate teacher-student interactions and involve limited student-student interactions. An advantage of xMOOCs is that they significantly broaden the number of students who can be exposed to university-level courses. xMOOCs focus on concise, targeted short videos rather than full-length lectures and use automated testing to check students' understanding as they work through the content.

The xMOOCs have attracted tremendous numbers of users and have played an increasingly important role in online learning. Millions of learners with different professional backgrounds and motivations came from different countries and gathered in the same “classroom”; therefore, higher education is broadened to an unprecedented worldwide range. According to the report from Columbia University [1], the most commonly identified goal for an institution to offer a MOOC is extending the reach of the institution to a wider audience and improving access to education. As is reported by Coursera, there are more than 8 million students who have enrolled in over 700 courses provided by 110 universities in Coursera. We can state that the rapid rise of MOOCs gives a good opportunity to explore globalized education problem.

However, together with large enrollment, it also brings another problem: low completion rates [2]. A team from University of Pennsylvania reported that the completion rate was only 4% by analyzing 16 courses in Coursera [3]. Based on the data given by a MOOCs statistics project (http://www.katyjordan.com/MOOCproject.html), the maximum completion rate in Coursera is less than 12%, and the average completion rate is only 6.06%. Though completion rate is not the only metric for learning in a MOOC, high completion rate will lead to more success for a MOOC course.

Many reasons can cause the low completion rate [4], such as insufficient time for learners and language barrier for nonnative speaking learners, or assume too much preknowledge for learners. Some researches show that forum users with less drop-out rate than not-forum users [5] and the drop-out probability decreases with more forum activities in forum users [6]. The motivation of this paper is to increase user activities by recommending study partners to target users. Furthermore, the recommendation will strengthen the interactions between learners and then facilitate solving problems by discussing with each other. Finally, it will help to improve the completion rate of xMOOCs.

With the revolution of Web 2.0, social networking is increasingly attracting the attention of academic and industry researchers. Social network analysis (SNA) is a technique analysis social network based on network theory. Recently, researchers in higher education field try to use social network analysis to help solve problems in higher education. These methods focused on how to improve teaching quality by importing social networking into traditional teaching classrooms or analyzing relationship between students in the same class. Chen et al. [7] studied student experience in learning process through the analysis on Twitter feedback about classroom teaching. Jiang et al. [8] stated that latent offline friendship between students who enrolled in the same course is an important relationship that can affect online discussions on course problems. Koutropoulos et al. [9] evaluated learners' participation feelings and needs by analyzing student feedbacks about MOOCs courses through Twitter. This is helpful for designing MOOCs platform. Griinewald et al. [10] presented that the communications between students in MOOCs have strong social collaboration features. Anderson et al. [11] classified participants into five types by analyzing the engagement of those users who join MOOCs classroom learning. Sinha [12] proposed a method to help collect curriculum issues from students automatically for course teachers in Coursera course forum. Brinton et al. [13] tried to predict the number of posts of a MOOCs course in the course forum through statistical analysis and proposed a generative model to infer the relevance between these posts and course content. Breslow et al. [14] pointed out that strengthening cooperation between learners will help improve students' scores.

The aim of this paper is to provide methods to recommend study partners for students in the same xMOOCs course. In general, if someone has a question about certain course concept, he/she can ask for help in the course forum, but the message that he/she posts may be covered by numerous posts so that it cannot be answered by someone else. The recommendation system will recommend someone who may be able to answer the question to him/her, and he/she can ask for help more specifically. In order to achieve this objective, we first established a behavior model to describe learner's behavior features by analyzing their forum activities such as posts and comments. Then a topic model with term dictionary was proposed to compute the similarity over topics among forum learners. Meanwhile, a social network among all learners was extracted by treating a conversation in the forum as a relationship edge. At last, we recommended study partners with high topic similarity and high relationship strength to the target learner. The recommendation will help to improve the chance to solve problems which learners encounter during the learning process and then keep their learning enthusiasm. The main contributions can be summarized as follows.

1. We introduce a method for classifying learners based on behavior features from the perspective of their activities in the course forum. Learners can be divided into three groups: like-questioning learner (*Q*-learners), like-answering learner (*A*-learners), and normal learner (*N*-learners).
2. We propose an approach based on latent Dirichlet allocation (LDA) model to measure the learners' course concept awareness. To make the topic similarity among learners computable, we construct a term dictionary to constrain the topic words based on the fact that the discussion topic in one course forum will not change dramatically.
3. We construct a social network according to the learners' activities in the course discussion forum and employ social network analysis to measure the strength of the relationship tie among learners.
4. We provide an in-depth analysis of the experiment results, revealing that making use of learner's behavior features, topic similarity, and relationship tie strength together can be of great benefit to recommend study partners for xMOOCs learners.

The rest of this paper is organized as follows. In Section 2, we review the previous work on friend recommendation. In Section 3, an overview of the proposed study partner recommendation system is described. The concrete approach of our recommendation system is presented in Section 4. In Section 5, we outline our experiment results on the datasets of Coursera. Finally, we conclude this paper and state several directions for future work in Section 6.

## 2. Friend Recommendation Method

Recommendation systems can be divided into two areas of focus: object recommendation and link recommendation [15]. To recommend study partners to a course learner is similar to recommend a friend to a user in social network. A “friends-of-friends” (FOF) method is one of the most widely used approaches in link recommendation. The method is useful and efficient due to ease of implementation and nature for humans to be drawn together through association [16, 17]. There are three main methods in the friend recommendation system.

### 2.1. Collaborative Filtering Based Recommendation

Collaborative filtering (CF) recommendation suggests items for people based on users who are alike with them. The “friends-of-friends” method can be seen as a way of adopting the ideas in collaborative filtering. However, the reason why one person adds a new friend is complicated. For example, one may accept a new friend because they study at the same school or they come from the same city or they have similar interests and so on. “Friends-of-friend” method may recommend friends to people with whom they are not similar and who may share information the user is not interested in. Bian and Holtzman [18] presented a CF-based friend recommendation system based on personality matching. Agarwal and Bharadwaj [19] proposed a CF-based framework that offers a list of friends to the user by leveraging on the preference of like-minded users, with a given small set of people that the user has already labeled as friends.

### 2.2. Graph Based Recommendation

The articulated social network structure can be considered as a social graph, and the task of recommending friends to a specific user is the same as predicting new links in this graph. Graph based approach made recommendation of friends by considering the local features [20] or global features [21] of graph and compared several local similarity measures, such as common neighbor, Jaccard's coefficient, shortest path, and Katz coefficient in the graph. Some researchers also proposed a method to predict users' residence locations based on users' social graph and profile [22]; this method can be further used for recommending friends who are like the target user.

### 2.3. Content Based Recommendation

Content-based recommendation method is based on the user's previous behavior or interests. User behaviors such as web browsing and interaction with other members often reflect user interest, as well as user self-generated content such as user profile, semantic tag, and posted messages. Manca et al. [23] presented an algorithm to infer users' interests form their bookmarking content and recommended friends with similar interest in the social bookmarking system. Wang et al. [24] presented a semantic-based friend recommendation system to discover life styles of users from user-centric sensor data, measured the similarity of life styles between users, and recommended friends to users if their life styles have high similarity. Cheung and She [25] analyzed the user's interests from the shared content, especially images with manually annotated tags and found similarities among people from the Bag-of-Features tagging. These content-based recommendations often established a user model to represent their interests and behaviors and estimated how different users are related to each other by matching the user models. And then recommend friends who with higher similarity to a specific target user.

In this paper, we combine content-based approach with graph-based approach. We first establish a user behavior model to represent user's behavior. Meanwhile, we also construct a relation network by users' interaction in the course forum. Then we incorporate the user behavior feature into the network and recommend friends for users based on similarity measures. Different from other friend recommendation systems, the relationship between learners is not an explicit friendship tie but only a latent communication in the course forum. The formation of this tie is more random, and the tie weight is weaker. On the other hand, to recommend a study partner for a learner we should pay more attention to specialty than diversity.

## 3. System Framework Overview

**Figure 1:**
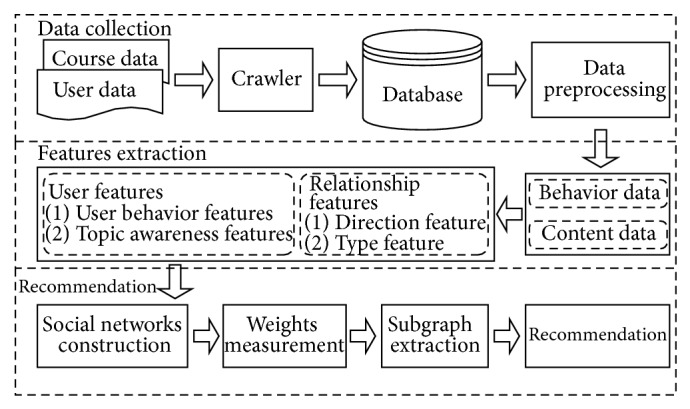
Proposed recommendation framework.

An overview of our proposed study partner recommendation system framework is outlined in Figure 1. The framework consists of three modules, which are data collection module, features extraction module, and recommendation module.

*Data Collection Module.* We collect the dataset from the biggest xMOOCs site, Coursera. The candidate content of data collection includes course data and user data.

(*1) Course Data. *The content of a specific course includes course name, course instructor, course categories, course duration, course starting and ending date, university offering the course, course location, and so forth. The most important content among the course data is all messages posted in the course discussion forum, including threads, posts, comments and their posted user, posted date, and posted text.

 (*2) User Data. *The user data is the content relative to a specific user in Coursera site such as user ID, user name, user role, and user location.

We save crawled data to database for preprocessing such as word segmentation and remove stop words and some other specific words such as “http://,” “www,” “href,” “com,” and “org.” 

*Feature Extraction Module.* We extract features from preprocessed data. The two major features extracted are listed as follows.

(*1) User Features.* We select the useful information from user's profile and user generated content and then propose a user behavior model to generate user behavior feature vector **f**_*B*_. We also propose a user topic model to generate user topic awareness feature vector **f**_*T*_. Therefore, the user features are represented by these three measures as follows:

(1) $$\begin{array}{c} UF_{u}=\left(f_{B},f_{T}\right). \end{array}$$

The detail of user behavior model and user topic awareness model will be described in Section 4.

(*2) Relationship Features. *The relationship between users is established while discussing each other. In this paper, we extract the relationship direction as well as relationship type.

*Recommendation Module. *After user feature vector and relationship features are extracted, we construct a relationship network among all active students who are involved in the course discussion forum. Discussion Forum in Coursera is more like a Q&A platform such as Yahoo Answers. However, the topics of discussions are more narrowed on courses but not daily conversation. A social network is formed through communication among those users in the forum. Every user in the same course can be treated as a node and one conversation represents an edge. However, the reason for the formation of an edge is not complex as other social networks. For example, in Facebook network, someone may find old friends from the same school or make new friends with someone who has similar interest or introduced by a common friend. In the Coursera Forum, the relationship between two nodes just represents a conversation. So it is a simplified social network which has narrowed topics.

## 4. User Model and Recommendation

In this section, we will introduce the specific approach of study partners recommendation, especially the user model and recommendation based on social graph.

### 4.1. User Model

#### 4.1.1. Candidate Users

**Figure 2:**
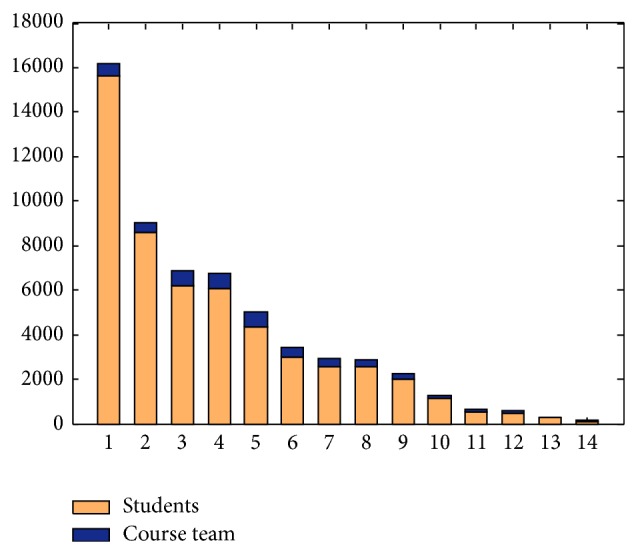
The weekly number of messages.

There are three different user roles in Coursera courses:* System Administrator* (including Coursera Staff and Coursera Tech Support),* Course Team* (including Instructor, Staff, and Community TA), and* Student*. The latter two groups are the major users in the course forum discussion. We illustrated the number of messages submitted by these groups every week in Figure 2. Course Team only answered fewer questions due to the limited employees though they are able to answer most questions. Moreover, the activities of Course Team group are not relevant to the activities of Student group. The activities of Student group are declining with more and more students quitting study while the activities of Course Team group are stable throughout the course process. It is because the course team has spent more time on video lectures and quiz so that they cannot spend lots of time on discussion forum activities. So we just take the student group as candidate pool for our recommendation.

**Figure 3:**
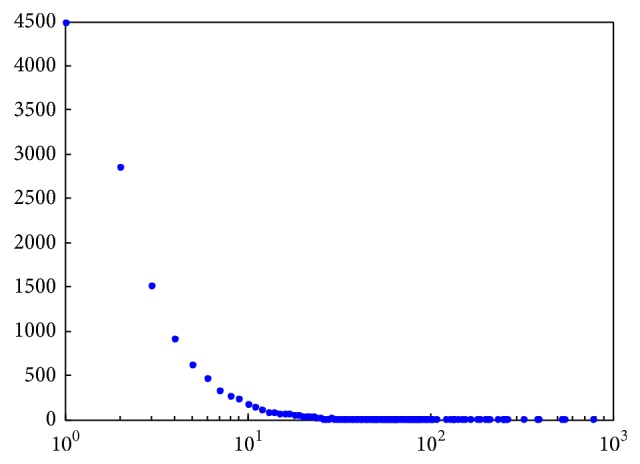
The number of messages versus the number of students.

In the 10 selected courses, a total of 53,662 messages (including posts and comments) were submitted by 13,064 students who participated in the forum discussion. However, as is shown in Figure 3, nearly 6,000 students submitted only one message. This part of the learners can be seen as nonactive learners. Research shows that the probability of dropout decreased dramatically for those learners who post one or more messages in the forum in first four weeks of the course [6]. Therefore, this paper selects those learners who post less than five messages in the course forum as target users and recommends study partners to them. On the other hand, reducing the recommendation pool will help to improve the efficiency of recommendation. Those learners who have never posted any message in the course forum are not within the scope of this paper because we cannot acquire their learning activities.

Therefore, active students in the course forum are candidate users for study partner recommendation.

#### 4.1.2. Messages for User Model

The messages posted in the course discussion forum can be roughly divided into three categories [13]. The first is the small conversation which is not relevant to course content (such as self-introduction or initiating a study group). The second is the questions about the course arrangements; for example, what is the due date of homework? How to download course materials?. The third is Q&A about the course-related content, so it is the most important and informative. In this paper, we only focus on messages belonging to the third category.

For convenience, course team often creates some subforums to organize all messages in the course discussion forum. Every subforum indicates what category of content is discussed. Therefore, we can simply label the category of every message in the forum by checking which subforum it belongs to.

#### 4.1.3. User Behavior Model

As we know, some students prefer to ask more questions while some students prefer to answer more questions. We propose the user model to represent user's behavior and classify users into three classes: *Q*-learners (prefer to ask questions), *N*-learners (normal class), and *A*-learners (prefer to answer questions). Obviously, recommending an *A*-learner student to a *Q*-learner student is a more effective recommendation and will maximize the learning effect for both students.

The message in the course discussion forum may be one of three types.

(*1) Thread*. It corresponds to a separated question. Someone can initiate a new thread if he/she has a new question to be solved.

(*2) Post*. It is a response to a thread; anyone can submit one or more posts to answer the question or to provide some other piece of information.

(*3) Comment*. It is just like post but is a response to a post. So a comment is organized under a post.

To identify if a message is a question or nonquestion, we just simply check whether there is question word such as “what,” “why,” and “which” or punctuation such as “?.”

As the author of a message, a user can initiate a new discussion thread, reply with a new post to an existing thread, or add new comment on posts. Therefore, the role of users can be divided into three types, namely, thread starter, poster, and commenter. Moreover, the same user can do all three activities.

Suppose a student *u* in a course forum posted *M*_*ui*_ messages; *i* is the message category where *i* = 1 when the message is a thread, *i* = 2 when the message is a post, and *i* = 3 when the message is a comment. And there are *Q*_*ui*_ messages which are labeled as questions in *M*_*ui*_ messages. Then we define the total number of messages in the course forum as *M*_*ai*_, and there are *Q*_*ai*_ messages which are labeled as questions. So we can define the student's question index *P*_*u*_:

(2) $$\begin{array}{c} P_{u}=\frac{1}{3}\overset{3}{\underset{i=1}{\sum }}\left[k_{i}\times \frac{Q_{ai}}{M_{ai}}\times {\left(\frac{Q_{ui}}{M_{ui}}\right)}^{2}\right], \end{array}$$

where *k*_*i*_ is a constant parameter between 0 and 1; all active students can be divided into three types by choosing appropriate *k*_*i*_ parameter and setting an appropriate interval for question index.

#### 4.1.4. User Topic Awareness

**Figure 4:**
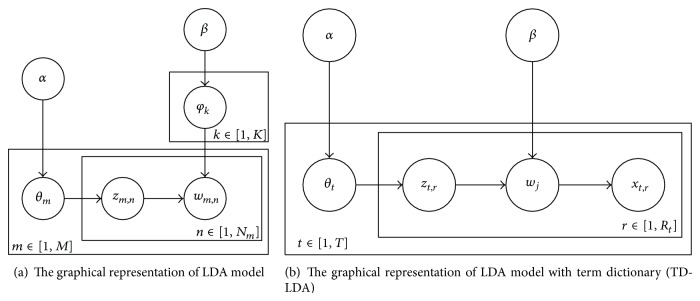
Classical LDA topic model and proposed topic model for recommendation study buddies.

To recommend learning partners for a target student, we should also consider the relevance of students on the course content in addition to the behavior characteristics of individual students. Therefore, we proposed a topic model to measure the similarity of students over topics.

latent Dirichlet allocation (LDA) is a widely used generative model to infer the latent topic distribution in a large corpus. All documents are represented as a “document-word” matrix and then are clustered into several different topics, and the distribution of each document over topics and the distribution of each topic over words are calculated finally.  Figure 4(a) is a graphical representation of LDA model.

Generally, topics in documents will evolve with new topic emergence; however, in xMOOCs courses, most learning materials are offered by course team and the topics are focused on fixed course concept. Therefore, the topics in the course forum also focus on limited course concepts, and topic words about every concept are stable. Therefore, we can construct a term dictionary for each course in advance and then infer the topic distribution of discussion in the course forum over these terms and deduce the topic distribution of every student over topic terms. The proposed topic model can be represented by a graphical model as Figure 4(b).

Suppose there are *T* threads in one course discussion forum, and there are *R*_*t*_ relationship edges among all involved students. *w*_*j*_ is the constructed topic term dictionary, and *z*_*t*,*r*_ is course topic, *θ*_*t*_ is the distribution of thread over course topic, *x*_*t*,*r*_ is the distribution of term dictionary over edges, and *α* and *β* are two hyperparameters. Thus, the generative process is as follows.

1. For all threads *t* ∈ [1, *T*], choose probability coefficients over topics *θ*_*t*_ ~ Dir(*α*).
2. For each of relationship edges *r* ∈ [1, *R*_*t*_] in thread *t*,
  - choose one of *k* topics *z*_*t*,*r*_ ~ Multinomial(*θ*_*t*_);
  - choose one of *D* term dictionary *w*_*j*_ from the multinomial distribution *p*(*w*∣*z*_*t*,*r*_, *β*) given by *z*_*t*,*r*_ and parameter *β*;
  - choose the edge *x*_*t*,*r*_ from the conditional distribution *p*(*x*_*t*,*r*_∣*w*_*j*_) defined by the generative term model of *w*_*j*_.

Here, different from the original LDA model, the relationship edge between two students is described as the distribution over term dictionary rather than a word. That is, every edge is generated by a mixed model of some discrete words. Given the parameters *α* and *β*, the joint distribution of observables and the hidden variables is

(3) pθ,z,w,x ∣ α,β =p(θ ∣ α)∏r=1Rpzr ∣ θpwj ∣ zr,βpxr ∣ wj,

where **z**, **w**, **x** are the vectors of course topic *z*_*t*,*r*_, topic term *w*_*j*_, and edge *x*_*t*,*r*_ in each thread message.

Our goal is to estimate the topic distribution conditional probability given by parameters *α* and *β*:

(4) $$\begin{array}{c} p\left(x\;|\;\alpha ,\beta \right)=\int \sum_{z}\sum_{w}p\left(\theta ,z,w,x\;|\;\alpha ,\beta \right)d\theta . \end{array}$$

Estimating this probability is intractable, and we follow variational inference to approximate the probability. So we define the following variational distribution for all topic dictionary words:

(5) p(θ,z,x ∣ α,β)=q(θ,z ∣ γ,ϕ)=q(θ ∣ γ)∏r=1Rq(zr ∣ ϕr),

where *γ* and *ϕ* are satisfied:

(6) $$\begin{array}{c} q\left(\theta \right)\sim \text{Dir}\left(\gamma \right),\\ q\left(z_{r}\right)\sim \text{Multinomial}\left(\phi_{r}\right). \end{array}$$

Take log on (4) to obtain a lower bound:

(7) log⁡p(x ∣ α,β)=log⁡∫∑zp(θ,z,x ∣ α,β)q(θ,z)q(θ,z)dθ=log⁡Eq[p(θ,z,x ∣ α,β)q(θ,z)]≥Eqlog⁡pθ,z,xα,β−Eqlog⁡qθ,z.

For simply, let

(8) lγ,ϕ;α,β=Eq[log⁡p(θ,z,x ∣ α,β)] −Eqlog⁡qθ,z.

According to original LDA model, we compute the derivative

(9) ∂l∂αi=Tψ∑l=1kαl−ψαi +∑t=1Tψγri−ψ∑l=1kγdl,

which can be solved with the Newton-Raphson algorithm. Thus, we can infer the topic distribution of relationship edges.

### 4.2. Recommendation

The topic similarity of two students is measured by the distance of their topic awareness. Combining topic similarity with the relationship matrix among all students in the same course, relationship network can be constructed. Furthermore, the recommendation can be fulfilled according to the weights among students.

#### 4.2.1. Social Network Construction

**Figure 5:**
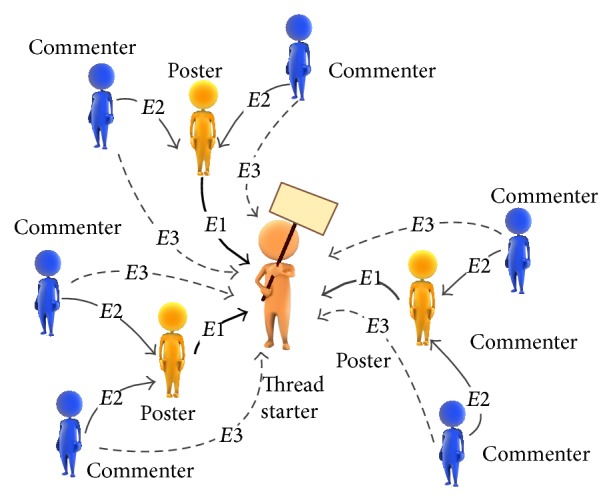
The relationships between the forum users.

In the course forum, each conversation not only transferred information from one user to another user, but also established a relationship edge between two users. According to the structure of course forum in Coursera, all the messages in the same thread focus on the same problem and contain many posts and comments. Therefore, there are edges between thread starter and all poster and commenter in the thread; there are also edges between posters and all comments in the same post. All probable edges in one thread are demonstrated in Figure 5. Among them, the edge from poster to thread starter (edge *E*1) and the edge from commenter to poster (edge *E*2) are direct edges, while the edge from commenter to thread starter (edge *E*3) is indirect one.

As previous analysis depicts, we can divide all students into three groups: *Q*-learner, *N*-learner, and *A*-learner. Therefore, there will be 27 different relationship edge types combining student behavior features and roles as shown in Table 1.

According to Table 1, a relationship type matrix *MT* can be constructed with its element being the representation of the relationship between students. And the value is the weight of each type. The dimension of the matrix is 3 × 3 × 3. There might be more than one type of relationships between two students. In our recommendation system, we prefer to recommend an *A*-learner to a *Q*-learner rather than recommend a *Q*-learner to another *Q*-learner even though both *Q*-learners have higher topic similarity. Therefore, the weight value of *E*1_*AQ*_, *E*2_*AQ*_, and *E*3_*AQ*_ is much greater than *E*1_*QQ*_, *E*2_*QQ*_, and *E*3_*QQ*_. The different activities features will also avoid an echo-chamber effect in learners.

Therefore, the relationship network between students can be represented by a weighted directed graph. Consider two neighbor nodes, *u* and *v*, the weight of the directed edge *uv* is given by the following formula:

(10) weightuv=α∏i=1lTa(u,v) +β∑j=13∑k=13∑l=13sj,k,lMTj,k,l.

The weight between two users consists of two terms. The first term is the topic awareness similarity between two nodes, that is, the product of topic awareness on all *T* words in topic term dictionary. The second term is the sum of all existing relationship weights defined by relationship type matrix *MT* between two nodes, where *s*_*j*,*k*,*l*_ is the number of relationships which belong to type (*j*, *k*, *l*) between two nodes. If one type relationship does not exist, the *s*_*j*,*k*,*l*_ is set to 0.

#### 4.2.2. Graph-Based Recommendation

**Figure 6:**
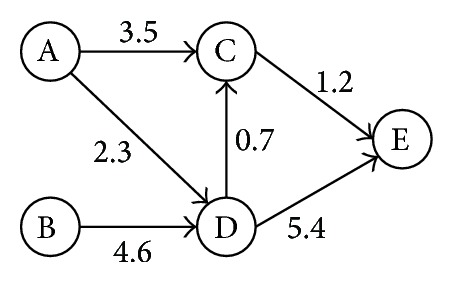
Example of a learner's network.

As noted above, the relationship among students is a weighted directed graph. Let us start with a simple network as shown in Figure 6.

In this graph, students C and D are direct neighbors of E, and students A and B are in-direct neighbors. Unlike FOF recommendation, although node E is connected by node A's both friends (C and D), user B is the best recommendation because of its strong connection with user D.

To find recommendations for target student *v*, we extract a subgraph containing all in-directions and the corresponding neighbors, and the neighbors of *v*'s neighbors are connected by all in-directions. Then the recommendation scores between all selected users and user *v* are calculated by

(11) $$\begin{array}{c} \text{Score}\left(uv\right)=\overset{P}{\underset{p=1}{\sum }}\overset{E_{p}}{\underset{e=1}{\prod }}\text{weight}\left(u_{ij}\right). \end{array}$$

The final score is the sum of all *P* paths between user *u* and user *v*, where the weight of every path is the product of weights of edges on the path.

## 5. Experiment Results

To validate our approach to the study partner recommendation problem in xMOOCs courses, we gathered data from the Coursera API. Basic information of all courses such as course name, start date, course category as well as forum messages of 15 courses which are in progress or just finished under “Statistics and Data” category are crawled and saved to a database. We tested our LDA with term-dictionary and corresponding algorithm against original LDA model.

### 5.1. Dataset Collection

Coursera provided application API to access its basic data by requesting some specific URLs, and JSON format data will be returned. We gathered three datasets: course information, user information, and forum messages information. The course information includes course ID, course name, course categories, instructor, open times, starting date, and duration. The user information includes user ID, nickname, gender, birth date, and user role. And the forum messages information includes message type (thread/post/comment), message ID, title, author ID, posted time, tags, and content as well as the hierarchy of these messages. Our data consist of 633 courses, 15 course forums, and over 10000 users involved in course forums. After that, we do some preprocessing work such as choosing 10 course forums in which the courses lasted more than 7 weeks or have finished and deleted inactive users. The final data information is as shown in Table 2.

### 5.2. Results

#### 5.2.1. User Behavior Classification

**Figure 7:**
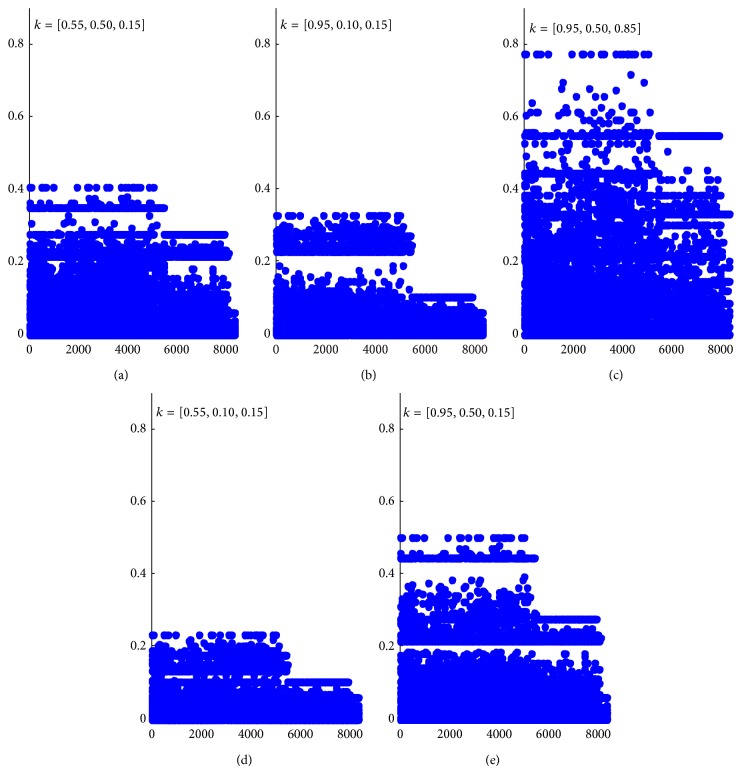
The question index of active students.

According to (2), by selecting different parameter *k*, the question index of all 8614 active students in 10 courses is shown in Figure 7. Different parameter *k* leads to different clustering result. In Figures 7(a), 7(b), and 7(c), students are clustered into two groups. In Figure 7(d), all students are belonging to same group. In Figure 7(e), students are clustered into three groups.

In this paper, we choose parameter *k*_*i*_ as [0.95,0.50,0.15] and set the interval as (12):

(12) $$\begin{array}{c} u=A\;\;P_{u}\in \left[0,0.2\right],\\ u=N\;\;P_{u}\in \left(0.2,0.4\right],\\ u=Q\;\;P_{u}\in \left(0.4,1.0\right], \end{array}$$

where *u* = *A* means that the student is more likely to answer questions, *u* = *Q* represent that the student is inquisitive to ask more questions, and *u* = *N* when the student is moderate to ask or answer questions.

#### 5.2.2. Recommendation Performance

In order to verify the validity and accuracy of the proposed method, we split each course forum messages into two parts according to posted time and then take the first part as training data while the second part as validate data. After training, we randomly select students from training data as target user and recommend study partners to him/her. If the recommended student communicates with target user in the second part data, the commendation is valid.

Suppose *N* target users are selected in the first part, and we recommend *m* study partners for each target user. For target user *u*_*n*_, there are *k*_*n*_ relationship edges between target user *u*_*n*_ and *m* recommended users. So the accuracy can be measured by

(13) $$\begin{array}{c} \text{Precision}=\frac{1}{N}\overset{M}{\underset{m=1}{\sum }}\overset{N}{\underset{n=1}{\sum }}\frac{k_{n}}{m}. \end{array}$$

**Figure 8:**
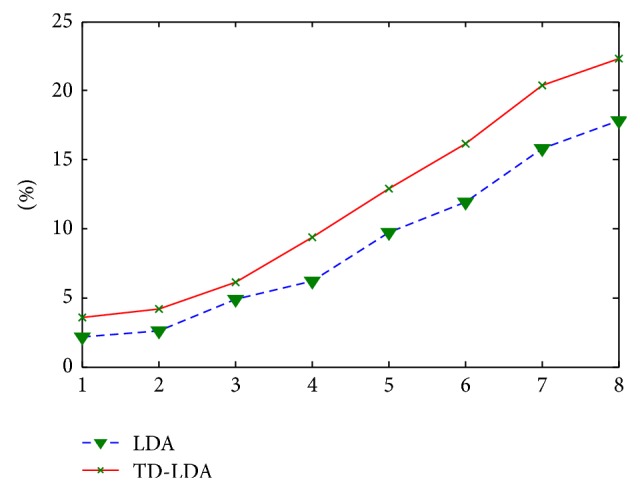
Comparison of recommendation precision between LDA and TD-LDA.

By changing the split time every week, we do the same recommendation experiment based on LDA model and LDA model with term dictionary (TD-LDA). The final average recommendation precision of 10 courses is shown in Figure 8.

In the first 3 weeks, the number of students is large while the forum activities are not sufficient, so the relationship matrix of students is very sparse and the behavior feature of students is not accurate. So the recommendation accuracy of both two methods is low and the difference is not significant. But with the time passed, the behavior feature is getting more accurate, and the relationship edges are increasing while the total number of students is decreasing because some students drop out of the course. The accuracy of the proposed method is much better than the tradition LDA although the accuracy of both two methods is increasing.

After the recommendation model was tuned up, we apply the model to recommend study partners for the rest 5 courses we have gathered from Coursera. We put learners into three groups by analyzing forum messages in first 4 weeks and randomly select 50 *Q*-learners. Then we recommend 20 study partners for every learner and compute recommendation accuracy according to (13). We calculate average accuracy by repeating the experiment 10 times for every course. The results are shown as Table 3.

In the rest 5 courses, the recommendation precision for all courses is above 10% except course A. This is because the number of active learners in this course is much less than other courses. There are only 418 active learners in course bigdata-edu-001 while there are over 800 active learners in other courses.

## 6. Conclusion and Future Work

We presented a method for study partner recommendation in xMOOCs courses to address the problem of how to help students to finish their learning process and improve the completion rate of xMOOCs courses.

By studying this problem with support from social networks analysis and topic modeling, our conclusion is that the LDA model with term dictionary is more effective in recommendation compared to the original LDA model as for topic modeling in messages discussed in xMOOCs course forum. In this paper, we developed a study partner recommendation system based on LDA model with term dictionary that produce quality and relevant friend recommendations in addition to providing insights into each individual's behavior feature and perception of course concept. The result has shown that the proposed approach has thus far outperformed the original LDA approach.

The study partner recommendation system still has much room for improvement. The primary issue leading to not high enough recommendation accuracy is due to the lack of more specific behavior data of students. We also cannot reach those students who have signed up a course but not leave any message in the course forum. Furthermore, there are many other reasons for a student to drop out from a course such as the gap between his expectation and reality. How to design a more personalized course based on the student's level is a bigger challenge.

## Figures and Tables

**Table 1:** The type of relationship between learners.

From	To		
	*Q*	*N*	*A*
*Q*	*E*1_*QQ*_	*E*1_*QN*_	*E*1_*QA*_
	*E*2_*QQ*_	*E*2_*QN*_	*E*2_*QA*_
	*E*3_*QQ*_	*E*3_*QN*_	*E*3_*QA*_

*N*	*E*1_*NQ*_	*E*1_*NN*_	*E*1_*NA*_
	*E*2_*NQ*_	*E*2_*NN*_	*E*2_*NA*_
	*E*3_*NQ*_	*E*3_*NN*_	*E*3_*NA*_

*A*	*E*1_*AQ*_	*E*1_*AN*_	*E*1_*AA*_
	*E*2_*AQ*_	*E*2_*AN*_	*E*2_*AA*_
	*E*3_*AQ*_	*E*3_*AN*_	*E*3_*AA*_

**Table 2:** Dataset information.

Number of courses	10
Number of threads	6684
Number of posts	36669
Number of comments	21879
Number of active students	8614

**Table 3:** Recommendation precision for the rest 5 courses.

Course	Accuracy
Big Data in Education	6.36%
Computational Methods for Data Analysis	12.53%
Financial Engineering and Risk Management Part I	10.47%
Computational Molecular Evolution	10.68%
High Performance Scientific Computing	11.73%
